# Disruption of mTOR and MAPK pathways correlates with severity in idiopathic autism

**DOI:** 10.1038/s41398-018-0335-z

**Published:** 2019-01-31

**Authors:** Eleonora Rosina, Barbara Battan, Martina Siracusano, Lorena Di Criscio, Fiona Hollis, Laura Pacini, Paolo Curatolo, Claudia Bagni

**Affiliations:** 1 Department of Biomedicine and Prevention, University of Rome Tor Vergata, Rome, Italy; 2 Department of Fundamental Neurosciences, University of Lausanne, Lausanne, Switzerland; 3 Department of Systems Medicine, Division of Child Neurology and Psychiatry, University Hospital of Tor Vergata, Rome, Italy; 4 Department of Biotechnological and Applied Clinical Sciences, University of L’Aquila, L’Aquila, Italy

## Abstract

The molecular signature underlying autism spectrum disorder remains largely unknown. This study identifies differential expression of mTOR and MAPK pathways in patients affected by mild and severe idiopathic autism. A total of 55 subjects were enrolled, of which 22 were typically developing individuals and 33 were patients aged between 3 and 11 years, with autism spectrum disorder. A detailed history, including physical examination, developmental evaluation, mental health history and autism diagnostic observation schedule were performed for each patient. Components of the mTOR and MAPK signalling pathways were analysed from peripheral blood at the protein level. Patients were then stratified according to their clinical phenotypes, and the molecular profiling was analysed in relation to the degree of autism severity. In this cohort of patients, we identified increased activity of mTOR and the MAPK pathways, key regulators of synaptogenesis and protein synthesis. Specifically, rpS6, p-eIF4E, TSC1 and p-MNK1 expression discriminated patients according to their clinical diagnosis, suggesting that components of protein synthesis signalling pathways might constitute a molecular signature of clinical severity in autism spectrum disorder.

## Introduction

Autism spectrum disorder (ASD) is characterized by high phenotypic heterogeneity, including deficits in social interaction and communication, as well as repetitive and unusual behaviours^1,2^. The aetiology of ASD is still largely unknown, although a complex genetic basis is present^3^. Some insights have been gained through the study of specific genetic disorders, such as fragile X syndrome (FXS) and tuberous sclerosis (TSC), two monogenic disorders characterized by a high incidence of ASD ranging from 25 to 60%^4–6^. FXS, the most common form of inherited intellectual disability, is caused by the loss or mutation of the fragile X mental retardation protein (FMRP)^7,8^. FMRP regulates several aspects of mRNA metabolism^9–11^. At synapses, FMRP regulates local protein synthesis at different levels^9^, with one of the characterized mechanisms based on the binding to CYFIP1 (cytoplasmic FMRP interacting protein 1) and eIF4E (eukaryotic translation initiation factor 4E)^12–15^. TSC is a dominantly inherited multisystem disorder characterized by the formation of hamartomas in different organs and the brain, caused by mutations in the *TSC1* or *TSC2* genes encoding hamartin and tuberin, respectively. TSC1 and TSC2 form a biochemical complex that inhibits the mammalian (also named mechanistic) target of rapamycin mTOR signalling pathway^16^. A large majority (85%) of children and adolescents with TSC display epilepsy, 50% cognitive disorders and ~30–40% ASD^16,17^.

Several forms of syndromic autism and intellectual disabilities (IDs), including FXS and TSC, are associated with mutations in genes that regulate protein synthesis and affect structure, transmission and plasticity of synapses^18^. Thus, it has been hypothesized that aberrant synaptic protein synthesis might contribute to ASD and other IDs that share ASD-like clinical features^19,20^. In the brain, mTOR and MAPK signalling pathways regulate synaptogenesis and local protein synthesis^21^. The MAPK pathway regulates neural progenitor biogenesis, learning and memory^22,23^ and mRNA translation, by phosphorylation of TSC2 and eIF4E via MAPK-interacting kinase 1 and 2 (MNK1 and MNK2)^14,24^. The MAPK pathway is upregulated in patients with syndromic ASD^25^. Furthermore, the deletion of the 16p11.2 locus, which includes the *MAPK3* (mitogen-activated protein kinase 3) gene, is associated with ASD^26,27^.

The mTOR kinase is part of two functionally distinct biochemical complexes: mechanistic target of rapamycin complex 1 (mTORC1) and mechanistic target of rapamycin complex 2 (mTORC2)^24,28^. mTORC1 affects protein synthesis by phosphorylation of eukaryotic translation initiation factor 4E-binding protein 1 (eIF4E-BP1) and p70 ribosomal protein S6 kinase (S6K1)^21,28^. The mTOR pathway is dysregulated in several human diseases, including cancer and diabetes^29,30^ as well as intellectual disabilities, such as FXS, Rett syndrome and ASD^30^. Thus, there is a wealth of evidence implicating mTOR and MAPK pathways, and ultimately protein synthesis, in syndromic ASD. Surprisingly, however, less is known about the function of these pathways in idiopathic ASD.

Here, we sought to characterize mTOR and MAPK pathways in children with idiopathic autism who had no other identifiable clinical syndromes. We investigated the expression of the above-described two pathways in peripheral blood mononuclear cells (PBMCs) of children with idiopathic autism and in typically developing individuals (TDI). In addition to performing a global analysis of patients versus TDI, we also analysed the molecular profile according to the clinical severity of ASD to identify a potential molecular signature of the disease severity.

## Materials and methods

### Subjects

This research was approved by the ethical committee of the University Hospital of Tor Vergata (Rome, Italy) and all participants provided written informed consent. Clinical data were gathered from 33 patients (27 males and 6 females) with idiopathic autism, recruited among those attending the Division of Child Neurology and Psychiatry, University Hospital of Tor Vergata (Rome, Italy). Patients ranged in age from 3 to 11 years, and met clinical criteria for a diagnosis of ASD, based on the diagnostic and statistical manual of mental disorders, fifth edition (DSM5)^31^. The diagnosis of ASD was also confirmed by using the autism diagnostic observation schedule (ADOS) test. Exclusion criteria included (1) neurodevelopmental disorders of known aetiology (as FXS, TSC, chromosomal abnormalities), (2) significant sensory or motor impairment, (3) significant medical conditions known to affect brain development, (4) low birth weight (< 2200*g*) or prematurity (<36 week of gestation).

The neuropsychological evaluation of children with ASD, involving development and cognitive assessment, was conducted by means of a diagnostic protocol, which included the administration of (1) the psychoeducational profile third edition (PEP-3)^32^ to determine the developmental level of young children with autism, (2) Leiter International Performance Scale-Revised (Leiter-R)^33^ to evaluate non-verbal-cognitive levels and (3) the autism diagnostic observation schedule (ADOS)^34^ for measuring the severity of autistic symptoms. The patient group was compared with a control group of 22 TDI, children and adolescents, ranging from 3 to 17 years of age (10 males and 12 females), who attended the outpatient clinic of the “Tor Vergata” University Hospital (Rome) for routine visits. The medical history of these children did not report evidence of any neurological or psychiatric disease. All children underwent a medical workup, including neurological examination, awake/sleep EEG, standard genetic analysis and auxological measurements. In addition, medical record examinations and parental interviews about the child history were performed. Consistency was required between parental information and medical records.

PEP-3: a standardised norm‐referenced scale to assess the development of communication and motor skills and the presence of maladaptive behaviours of children suspected of ASD, with a developmental age between 2 and 7 years, who may be non‐verbal, have limited attention skills and poor concentration, and who are not used to a formal testing situation. The PEP-3 does not provide a development quotient (DQ) like PEP-R, obtained by dividing the child’s age-equivalent score by his/her chronological age. We generated the scale by estimating a development level (DL) by the ratio of developmental age/chronological age^35^ for each developmental subtest.

Leiter-R: expressively non-verbal measure of global intelligence with fair cross-cultural applicability. Its engaging, non-verbal format makes it ideal for use with individuals with ASD. It provides an IQ score, as well as percentile and age-equivalent scores for each subtest. For cases where the IQ score was not possible to evaluate, DL was assessed.

In accordance with a recent study, DLs of each subtest of the PEP-3, can be compared with Leiter-R IQ and can be considered as indicators of cognitive functioning in subjects with ASD^36^. The decision to administer the PEP-3 or the Leiter-R scale or both, was based on the child’s developmental skills, attention skills and age.

ADOS: a semi-structured assessment of communication, social interaction and relatedness, play, imagination and stereotyped or repetitive behaviours. This measure yields scores in the social domain, communication domain, as well as a combined score. A child can be classified as having autistic disorder, pervasive development disorder not otherwise specified (PDD-NOS), or as non-autistic.

The modules provide social-communicative sequences that combine a series of unstructured and structured situations. The examiner selects the module that is most appropriate for a child on the basis of his/her expressive language level. Module 1 is intended for individuals who do not have verbal language or use just simple words; Module 2 for individuals with some phrase speech who are not verbally fluent; Module 3 for verbally fluent children.

CSS (Calibrated Score System): a standardized severity score based on codes within the domains, that can be calculated to compare autism symptoms across modules^37^. Based on the CSS, each child was assigned to AUT, for severe symptoms, ASD, for mild symptoms and NS for non-significant symptoms. In our clinical severity analysis, we separated patients into “Mild” and “Severe” groups based on their CSS designation as “ASD” or “AUT”, respectively. The division into groups based on cognitive function was performed using the scores obtained by the cognitive evaluation Leiter-VR or development assessment PEP-3, in accordance with recent data^36^.

### Peripheral blood mononuclear cell (PBMCs) isolation

Blood samples were collected by venepuncture into EDTA-coated tubes. PBMCs were isolated from whole blood (9 ml) using Histopaque-1077 (Sigma-Aldrich) according to manufacturer’s instructions. All samples in this study represent patient peripheral blood mononuclear cells (PBMCs) that were freshly purified after blood collection and not cultured.

### Protein extraction and western blotting

Total protein extracts from PBMCs were obtained from the cellular pellet directly in 2X Laemmli Buffer. Samples were boiled and separated by SDS–PAGE electrophoresis and transferred to a PVDF membrane (GE Healthcare). Total and phospho-protein levels were detected simultaneously on the same membranes by using antibodies specific to the desired protein. Membranes were incubated using the following antibodies: rabbit anti-FMRP (1:1000, custom made by 21st Century), mouse anti-mTOR (1:500, CST #4517), rabbit anti-p-mTOR (Ser2448) (1:500, CST #2971), rabbit anti-TSC1 (1:1000, CST #4906), rabbit anti-TSC2 (1:500, CST #4308), rabbit anti-rpS6 (1:1000, CST #2217), rabbit anti-p70S6K1 (1:500, CST #9202), mouse anti-p-p70S6K1 (1:500, CST #9206), mouse anti-eIF4E (1:1000, Santa Cruz #SC-271480), rabbit anti-p-eIF4E (Ser209) (1:500, CST #9741), rabbit anti-4E-BP1 (1:1000, CST #9644), rabbit anti-p-4E-BP1 (Thr37/46) (1:500, CST #2855), rabbit anti-Rheb (1:500, Thermo Fisher #PA5-20129), rabbit anti-MNK1 (1:1000, CST #2195), rabbit anti-p-MNK1 (Thr197/202) (1:500, CST #2111), mouse anti-ERK1-2 (1:1000, Abgent #AM2189B), rabbit anti-p-ERK1-2 (Thr202/Tyr204) (1:500, Santa Cruz #SC-16982), mouse anti-GAPDH (1:2000, Thermo Fisher Scientific #MA5-15738). The following secondary antibodies were used: anti-rabbit or anti-mouse HRP, anti-rabbit or anti-mouse IgG Dylight 800 (1:10000 Promega #W4011, # W402B, and 1:2500, Thermo Fisher Scientific #SA5-35571, #SA5-35521, respectively) and anti-mouse or anti-rabbit IgG Dylight 680 (1:1000, Thermo Fisher Scientific #35518, #35568). Proteins were revealed using an enhanced chemiluminescence kit (Bio-Rad) and the imaging system LAS-4000 mini (GE Healthcare Life Sciences) or, for Dylight-conjugated secondary antibodies, the Odyssey Infrared Imaging System (LI-COR Bioscience). Total and phosphoproteins were revealed using the LI-COR system using differential fluorescently-tagged secondary antibodies to reveal modified and non-modified proteins on the same membrane without any overlap or stripping. All phosphoproteins were normalised relative to the total protein on the same blot. Total proteins were normalised to the average of both Coomassie staining of the membranes (control for total proteins on the membrane) and GAPDH signals. This normalisation is reported in arbitrary units (A.U.) in the figures. ImageQuant TL software was used to quantify the signals. Some antibodies revealed more than one signal for proteins. Specifically, ERK1-2 and p-ERK1-2 were quantified considering the two detected isoforms together. The antibody for rpS6 detected both the manufacturer-specified band at 33 kDa in addition to an unpredicted, non-specific band. In this case, we only quantified the single band at 33 kDa (the upper one) and the non-specific band was not included. The analysis of each protein represents the average of at least two technical replicates per subject.

### Statistical analysis

Sample sizes were based on our preliminary results, where we determined the need for a sample size of at least *n* = 21/group for a power of 85%, alpha = 0.05, and effect size: Hedges' g = 0.67. Randomization of subjects was not possible in this study, as the goal was to compare patients versus controls. Comparisons between patients with ASD and controls were performed using nonparametric Mann–Whitney U test. When more than two groups were compared, analyses were performed using generalized linear models with maximum likelihood estimation followed by post hoc Tukey’s multiple comparison test. InStat statistical software (GraphPad Softwares Inc., La Jolla, CA) was used for the statistical analysis. Canonical discriminant analysis (CDA) was used to investigate whether a set of differentially expressed proteins could discriminate among three groups of patients according to their clinical disease status (controls, mild ASD and severe ASD), using SPSS (IBM Corp. Armonk, NY, US) software, version 24.0. The analysis employed a stepwise technique, based on the Wilks’ ʎ test with the standard probabilities of *F* (3.84 to enter and 2.71 to be removed) for variable selection, keeping variables with a statistically significant classification performance (*p* < 0.05) in the model. Cross-validation was performed using a leave-one-out method to assess the model performance.

Significance was denoted as *p < 0.05, **p < 0.01, ***p < 0.001, ****p < 0.0001. Error bars represent the standard error of the mean (SEM). Variances between groups were similar. Outliers were calculated using the Grubbs’ outlier test and were excluded from the figures and the statistical analysis.

## Results

### Clinical summary

Comprehensive clinical data were gathered from 33 patients with idiopathic autism (Table 1).

**Table 1:** Clinical summary of the children with ASD

ID patient	Gender	Age^a^	IQ	DL	ADOS module	CSS	ADOS classification	Other features
3	M	10	60		3	9	AUT (severe)	GI, sleep disorders
4	M	11	69		3	10	AUT (severe)	–
5	M	9		0.59	2	6	AUT (severe)	–
10	M	8	90		1	6	AUT (severe)	–
11	M	8		0.40	1	6	AUT (severe)	ADHD, food selectivity
12	M	6		0.36	1	4	ASD (mild)	Celiac disease, food selectivity
13	M	10		0.59	1	4	ASD (mild)	Celiac disease
14	M	7		0.55	1	6	AUT (severe)	Sleep disorders, food selectivity, ADHD
15	M	6		0.82	1	1	NS	ADHD
17	M	8	89		3	6	AUT (severe)	GI disorders
18	F	5		0.43	1	6	AUT (severe)	ADHD
19^b^	F	8		0.39	Patient did not complete the assessment			ADHD
20	M	7		0.64	1	6	AUT (severe)	Sleep disorders, food selectivity
21	F	6		0.62	1	4	ASD (mild)	–
22	M	3		0.67	1	4	ASD (mild)	–
24	M	4		0.32	1	5	ASD (mild)	Sleep disorders
26	F	3		0.70	1	4	ASD (mild)	–
27^b^	M	3		0.83	Patient did not complete the assessment			–
28	M	7		0.27	1	7	AUT (severe)	Sleep disorders, food selectivity
29	M	10	90		3	6	AUT (severe)	–
30	M	5		0.42	1	7	AUT (severe)	–
31	M	8	92	0.95	3	4	ASD (mild)	Food selectivity
32	F	6		0.40	1	6	AUT (severe)	–
34	M	4		0.79	1	6	AUT (severe)	Sleep disorders, food selectivity
35^b^	M	8	72	0.44	Patient did not complete the assessment			–
36	F	4		0.53	1	7	AUT (severe)	
37	M	6		0.30	1	6	AUT (severe)	GI disorders
38	M	6	96		2	9	AUT (severe)	Immune dysfunction, food selectivity
39	M	10			1	4	ASD (mild)	Food selectivity
40	M	4		0.54	1	10	AUT (severe)	Sleep disorders, food selectivity
41	M	3		0.67	1	7	AUT (severe)	Sleep disorders, food selectivity
42	M	9	119		3	6	AUT (severe)	–
43	M	4		0.60	1	6	AUT (severe)	Immune dysfunction, food selectivity

*ADOS* autism diagnostic observation schedule, *CSS* calibrated score system, *IQ* intelligence quotient, *DL* development level, *GI* gastrointestinal

^a^Refers to the age of the subject at the time of blood puncture

^b^Patients who were unable to complete the neuropsychological assessment due to family reasons

At the time of the evaluation, 21 children had acquired verbal language. Of these, 9 children were able to use phrase speech, while the remaining 12 were able to use only simple words. Five children showed comorbid hyperactivity and attention problems, and two children were suffering from celiac disease. All patients had an established diagnosis of ASD at the time of enrolment. For this study, they were re-evaluated for core clinical features of ASD using the ADOS; for developmental skills using standardised scale psychoeducational profile third edition (PEP-3); and for nonverbal-cognitive abilities using the Leiter-R scale. Three patients were unable to complete the neuropsychiatric evaluations (Table 1). Their data were included in the global analyses, but excluded in analyses based on disease severity.

Thirty subjects were evaluated for the presence of ASD symptoms using the ADOS. According to the expressive language level of the child, the examiner selected the most appropriate ADOS module. ADOS-module 1 was used for 22 patients (10 non-verbal children; 12 verbal children whose language was constituted by simple words); ADOS-module 2 (for individuals with some phrase speech not verbally fluent) for two children; ADOS-module 3 (for verbally fluent children) for six patients.

All subjects, except one (ID 15), exceeded cut-off criteria scores for ASD on the ADOS. For each child, a score was calculated according to the Calibrated Score System (CSS), a standardized severity score based on codes within the domains, calculated to compare autism symptoms across ADOS-modules^37^. Each child was then assessed for cognitive and development. The PEP-3 or the Leiter-R scales or both were applied based on the child’s developmental skills, attention skills and age. Children were grouped according to the scores obtained in the cognitive evaluation Leiter-VR or functional assessment PEP-3, in accordance with recent data^36^ (Table 1).

### Expression of the components of mTOR and MAPK signalling pathways in patients with idiopathic autism

**Fig. 1:**
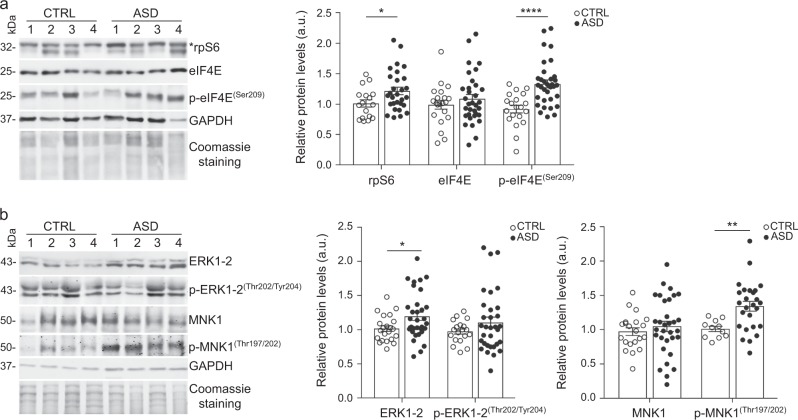
Protein levels in PBMCs of patients with non-syndromic autism and controls. **a** Left, representative Western blot showing protein levels of rpS6, eIF4E and p-eIF4E (Ser209). The molecular weight of each protein is indicated in kDa. Right, bar plots representing the quantification of the technical replicates where rpS6 and p-eIF4E levels are increased in ASD patients compared with controls (rpS6: *U* *=* 150, *p* *=* 0.03; p-eIF4E: *U* = 115, *p* *<* 0.0001). Error bars represent the standard error of the mean (*n* = 11-22 CTRL, 26-33 ASD; *p < 0.05, **p < 0.01, ***p < 0.001, ****p < 0.0001; Mann–Whitney U tests). Each dot represents the average of at least two technical replicates per subject. Total proteins were normalised to the average of Coomassie staining and GAPDH. Phosphoproteins were normalised for respective total protein levels. *indicates the quantified signal **b** Left, representative Western blot showing protein levels of ERK 1-2, p-ERK 1-2 (Thr202/Tyr204), MNK1 and p-MNK1 (Thr197/202). The molecular weight of each protein is indicated in kDa. Right, bar plots representing the quantification of the technical replicates, where ERK1-2 and p-MNK1 levels are increased in ASD patients compared with controls (ERK1/2: *U* *=* 242.5, *p* *=* 0.04; p-MNK1: *U* = 58.5, *p* *=* 0.004)

We first performed a global analysis of the expression levels of 17 proteins involved in the mTOR and MAPK pathways in the blood of patients with ASD compared to typically developing controls (Fig. 1 and Supplementary Fig. 1). We targeted proteins with a strong link to local protein synthesis modulation.

The key effector mTOR did not show any significant differences either in the total level or in the phosphorylation status (Ser2448 site) between ASD patients and controls (Supplementary Fig. 1a). The two downstream mediators of the mTORC1 complex activity, 4E-BP1 and the p70S6K1 kinase, were also similar between groups (Supplementary Fig. 1a,b). Moreover, no differences were observed in the translational repressor FMRP (Supplementary Fig. 1b). Interestingly, the 40S ribosomal subunit component, rpS6, was increased in ASD patients compared to controls *(p* *=* 0.03*)* (Fig. 1a). Moreover, while the translation initiation factor eIF4E did not exhibit differential expression between ASD patients and TDI, its activated phosphorylated form (Ser209) was significantly higher in ASD patients compared to TDI (*p* *<* 0.0001) (Fig. 1a).

Since mTORC1 activity and protein synthesis initiation are inhibited by the TSC complexes^38^, we next investigated if TSC1 and TSC2 levels were dysregulated in ASD patients. However, no differences were observed in TSC1 and TSC2 protein levels (Supplementary Fig. 1c). We also investigated the expression level of RHEB, a protein known to interact and activate mTORC1^38^. RHEB levels also did not differ between ASD patients and TDI (Supplementary Fig. 1c).

ERK1-2 (extracellular signal-regulated kinase 1-2) signalling is also involved in the translational control downstream of the activation of cell surface receptors^23,39^. The expression of p44/42 MAPK (ERK1-2) was significantly increased in ASD patients compared to controls (*p* *=* 0.04), however there were no differences in its phosphorylated status on the Thr202/Tyr204 residues (p-ERK1-2) (Fig. 1b). Interestingly, while the downstream effector of ERK1-2, MNK1, did not show differences between the groups, we observed a significant increase in MNK1 phosphorylation levels (Thr197/202) in ASD patients compared with controls (*p* *=* 0.004) (Fig. 1b), suggesting increased activity at the end of this pathway.

Taken together, our findings point to increased activity in mTOR and MAPK pathways in children with idiopathic ASD.

### Clinical-molecular correlations

**Fig. 2:**
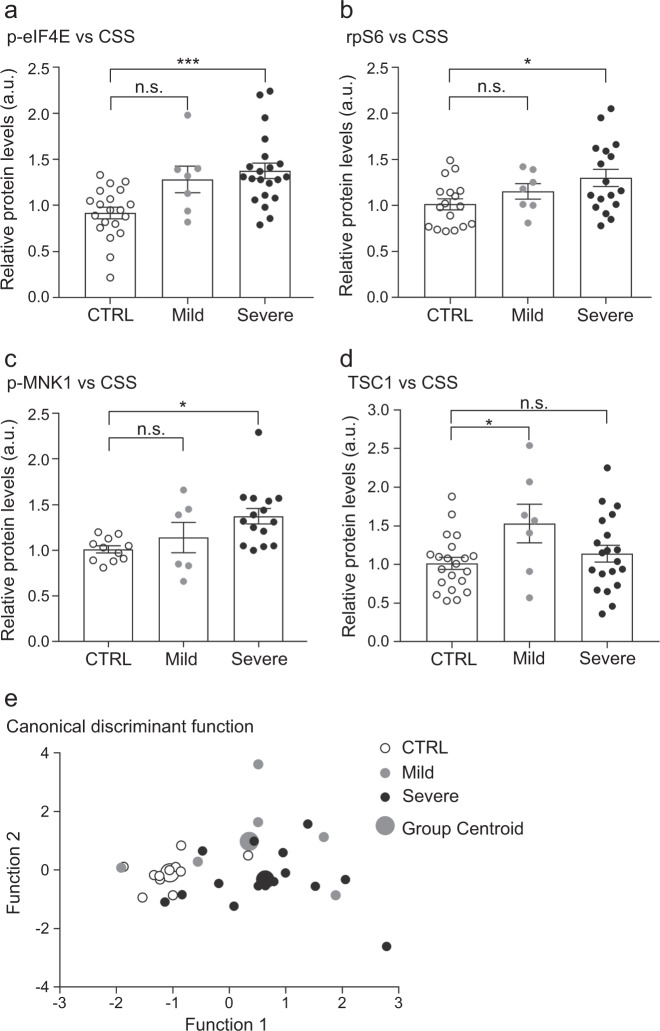
Protein levels in PBMCs correlate with the severity of the clinical phenotype. Bar plots representing the quantification of the technical replicates in the three subgroups, CTRL, Mild ASD and Severe ASD show significantly increased levels of **a** p-eIF4E (F(2,45) = 9.51; *p* *=* 0.0004) **b** rpS6 (F(2,38) = 3.70; *p* *=* 0.03) and **c** p-MNK1 (F(2,29) = 5.02; *p* *=* 0.01) in the severe subgroup compared to controls, while **d** TSC1 levels were significantly increased in the mild group only (F(2,45) = 3.24; *p* *=* 0.048). Error bars represent the standard error of the mean (*n* = 11-21 CTRL, 6-7 Mild ASD, 11-20 Severe ASD; *p < 0.05, **p < 0.01, ***p < 0.001; one-way ANOVA). **e** Scatter plot of the two canonical functions containing four proteins (p-eIF4E, rpS6, TSC1, p-MNK1) that discriminated subjects according to their severity into three groups: CTRL, mild ASD and severe ASD (Wilks’ Lambda = 0.480; Chi-square = 20.2; df = 8; *p* *=* 0.01). The mean discriminant scores for each group are depicted as group centroids. Four patients were not included in this analysis (see Table 1)

Given the heterogeneity of phenotypes found in autism spectrum disorder, and the variability of protein expression observed within our cohort, we hypothesized that protein expression may change in relation to the severity of the disease. To address this question, we analysed the molecular profile according to the clinical phenotype for each patient. Based on the CSS classification, we identified several of the previously analysed 17 proteins that correlated with the severity of the disease. Among those, p-eIF4E, rpS6 and p-MNK1 proteins, which were increased in the global analysis, exhibited significantly increased levels in the severe group compared with controls (p-eIF4E: *p* *=* 0.0003; rpS6: *p* *=* 0.03; p-MNK1: *p* *=* 0.01; Fig. 2a–c). While ERK1-2 levels were changed in the global analysis, we did not observe any correlation with the severity of ASD. Interestingly, we observed an increase of TSC1 levels in the mild ASD subgroup but not in the severe subgroup (*p* *=* 0.048; Fig. 2d).

Together, these findings suggest that mild and severe autism may be differentiated according to different protein expression profiles.

Finally, as we identified four proteins (p-eIF4E, rpS6, TSC1 and p-MNK1) that demonstrated an association with the clinical severity, we wanted to investigate if, together, those proteins could constitute a molecular signature for autism severity. Therefore, we performed a canonical discriminant analysis between the disease groups and healthy controls. Remarkably, the analysis generated two canonical functions that significantly separated the patient groups with 71.9% accuracy (*p* *=* 0.01; Fig. 2e). A cross-validation of the analysis revealed a robust model with 68.8% of cross-validated cases correctly classified. Thus, these four proteins are able to distinguish, with high accuracy, patients with ASD from typically developing controls as well as patients with mild versus severe autistic clinical features.

## Discussion

The complexity and heterogeneity of ASD clinical phenotypes makes it difficult to determine the severity of ASD symptomology, predict prognoses and stratify individuals for research purposes^40^. In this study, we investigated mTOR and MAPK signalling pathways in the peripheral tissue of autism spectrum disorder patients in order to identify a molecular signature both for the disease and for ASD severity. Globally, we found a significant increase of rpS6, p-eIF4E, ERK1-2 and p-MNK1 levels in patients with ASD compared with controls (Fig. 1). Interestingly, we observed a large variability in the protein expression levels of ASD patients compared with typically developing controls that was related to their clinical phenotype. When patients were stratified according to their CSS classification, we found several proteins (p-eIF4E, rpS6, TSC1 and p-MNK1) that appear to constitute a molecular signature of severity. Indeed, a canonical discriminant analysis revealed that p-eIF4E, rpS6, p-MNK1 and TSC1 proteins could discriminate between the three subgroups (controls, mild ASD and severe ASD) (Fig. 2e). We suggest that this set of proteins is a molecular readout, which correlates with ASD severity.

The identified proteins are positive translational regulators, suggesting a peripheral hyperactivation of protein synthesis in autism. Compelling evidence in mice and humans indicates that aberrant synaptic protein synthesis is associated with several forms of neurodevelopmental disorders^19,41–43^. At the molecular level, a dysregulation of MAPK and mTOR pathways has been suggested to disrupt protein synthesis^19–21,44–46^. Additionally, a link between two direct regulators of protein synthesis, eIF4E-BPS and S6K1 kinase, in autistic-like phenotypes in mice^20^ and human cells^46,47^ was proposed. Moreover, our findings are supported by studies in post-mortem brains and T cells from idiopathic ASD patients, where a hyperactivation of the mTOR pathway was observed^47,48^.

Functionally, in the brain, mTOR and MAPK are downstream effectors of group I mGluRs, NMDA and TrkB receptors^24^. MAPKs modulate the activation of MNK1, which phosphorylates eIF4E on serine 209^49^ and enhances protein synthesis^50^. Notably, our cohort of ASD patients displayed a significant increase of p-MNK1 and p-eIF4E, both in the global expression and in the clinical correlations (Figs. 1a, b and 2a, c). Surprisingly, the increase in p-MNK1 in ASD patients was not accompanied by an increase in ERK1-2 activation, suggesting that the activation of MNK1 is ERK-independent. MNK1 can also be regulated by the upstream MAP kinase p38^51^, thus, future studies should examine the possibility for increased activation of this MAP kinase. Furthermore, we analysed the phosphorylation of 4E-BP1 and did not see any significant differences between groups (Supplementary Fig. 1a), suggesting that the upregulation of eIF4E phosphorylation is linked to the MNK axis rather than the mTOR signalling. Ultimately, our findings indicate a hyperactivation of the MAPK pathway leading to hyperphosphorylation of p-eIF4E, which arguably results in aberrant protein synthesis.

Studies in humans and mice indicate that an increased activity of eIF4E leads to ASD and autistic-like phenotypes respectively^52–55^ eIF4E activity is modulated by the mitogen-activated protein kinase (MAPK)-interacting kinases (MNKs)^49^. Specifically, MNK1 is required for translational activation in cell migration, cancer metastasis^56^ and in response to BDNF in the mouse cortical neurons and in the dentate gyrus^14,57^. Our work here identifies a new role for p-MNK1 in idiopathic ASD, providing further evidence for the contribution of the MAPK pathway to idiopathic ASD^58^.

Patients affected by FXS and TSC disorders meet the diagnostic criteria of ASD, suggesting that mutations in these pathways may cause ASD^21,59^. The TSC complex inhibits mTOR activity, leading to translational repression^38,60^. *Tsc1* deletion in neurons has been linked to macrocephaly and embryonic lethality, and *Tsc1* models present defects in synaptic plasticity^61–63^. Despite the fact that the small sample size limits interpretations, the observed increase of TSC1 levels may indicate an increased inhibition of protein synthesis in the mild subgroup only. Moreover, these results suggest a possible bidirectional disruption of protein synthesis in ASD. A bidirectional alteration in protein synthesis has been previously proposed in two mouse models for ASD. *Fmr1*^*-/y*^ and *TSC2*^*+/-*^ mice displayed opposing effects in synaptic protein synthesis that compensate each other in the double mutant *Fmr1*^*-/y*^ *TSC2*^*+/-*^ mice^19^. While we observed increased levels of some positive regulators of protein synthesis in the severe subgroup, our data also found an increased level in the negative protein synthesis regulator, TSC1, in the mild ASD group (Fig. 2d). Our study strengthens the bidirectional disruption hypothesis, and indicates that similar mechanisms may occur in humans.

Our findings suggest an overlap in the molecular mechanisms between syndromic and idiopathic ASD. Recently, we demonstrated an increase in protein synthesis levels in non-neuronal cells of individuals with FXS^64^. As similar findings were obtained in neuronal and non-neuronal cells within the same mouse^64^, we suggest that peripheral levels of protein synthesis may function as a representative neuronal molecular signature for future clinical trials aiming to restore translational control in FXS and other neurodevelopmental disorders. Interestingly, several studies have explored the use of human peripheral blood cells as a surrogate for the neuronal molecular signature in neurological diseases, suggesting that lymphocytes may constitute an easily accessible “neural probe” to study neuropsychiatric disorders such as autism^65,66^. Overall, our results suggest that defective protein synthesis may constitute the potential molecular mechanism underlying ASD pathology across different etiologies^67^.

**Fig. 3:**
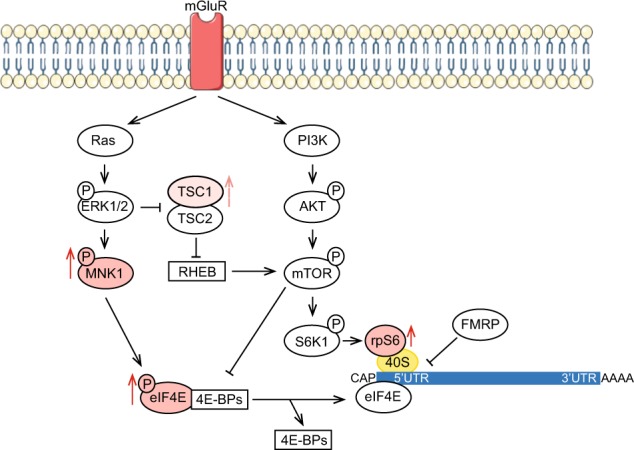
Upregulated pathways leading to excessive protein synthesis in the context of severe ASD. p-MNK1, p-eIF4E, TSC1 and rpS6 discriminate mild and severe ASD. Color code indicates different pattern of expression across the two ASD groups. Dark pink denotes upregulated proteins in severe ASD (p-MNK1, p-eIF4E and rpS6). Light pink denotes upregulated TSC1 in mild ASD. The dysregulation of each of those proteins can lead to an excessive protein synthesis

Our data propose that an increase in both MAPK and mTOR pathways possibly leads to aberrant protein synthesis (Fig. 3) in subjects with ASD, indicating that alteration of mTOR and the MAPK pathways may contribute to intellectual disabilities and to both syndromic and non-syndromic ASD. Although further studies in different cohorts are required, we suggest that measurements of key protein synthesis regulators may constitute a molecular signature in PBMCs that may be predictive for early diagnosis of autism, particularly across severity groups. This may facilitate the identification of suitable targets for future personalised therapies.

## Supplementary information

Suppl material

Suppl figure legends
