# Supplementary figures and images for: Disruption of mTOR and MAPK pathways correlates with severity in idiopathic autism
Source: Transl Psychiatry. 2019 Jan 31;9:50. doi: 10.1038/s41398-018-0335-z (PMC6355879; doi:10.1038/s41398-018-0335-z)

# Supplementary Figure 1

a

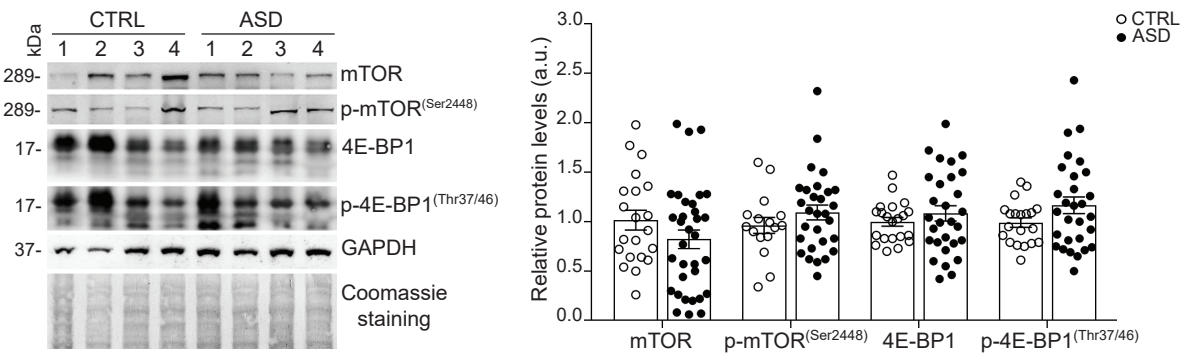

b

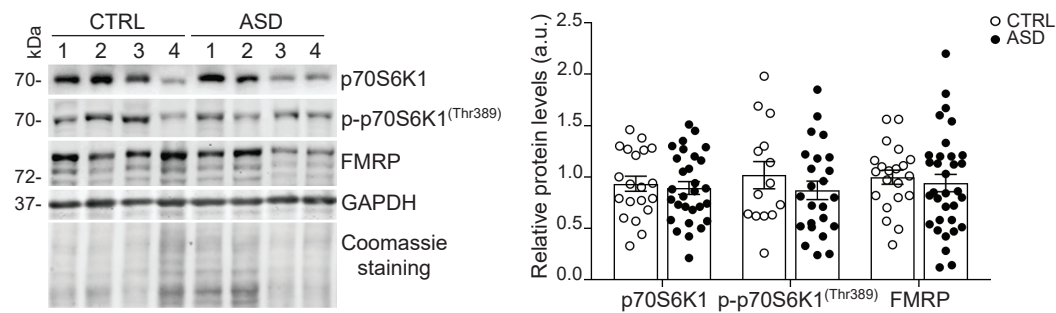

c

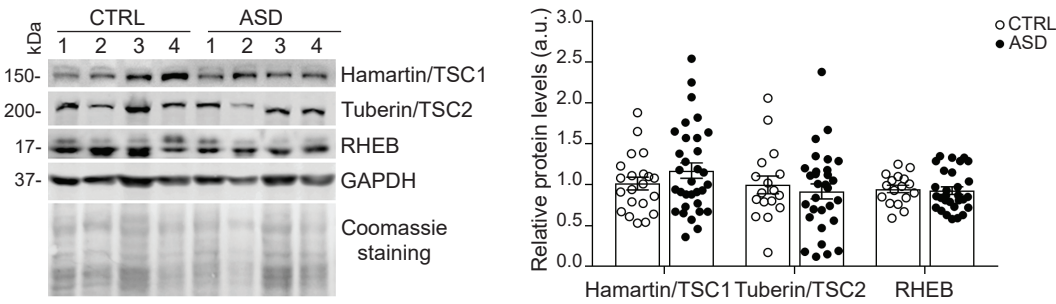

Supplement: Supplementary file 1 — Suppl material [file 41398_2018_335_MOESM1_ESM.pdf]
