# Supplementary material for: Disruption of mTOR and MAPK pathways correlates with severity in idiopathic autism
Source: Transl Psychiatry. 2019 Jan 31;9:50. doi: 10.1038/s41398-018-0335-z (PMC6355879; doi:10.1038/s41398-018-0335-z)
Supplement: Supplementary file 2 — Suppl figure legends [file 41398_2018_335_MOESM2_ESM.docx]

**Supplementary Figure Legend**

**Supplementary Figure 1. Protein levels in PBMCs of patients with non-syndromic autism and typically developing individuals.** (**a**) Left, representative Western blot showing protein levels of mTOR, p-mTOR (Ser2448), 4E-BP1 and p-4E-BP1 (Thr37/46). The molecular weight of each protein is indicated in kDa. Right, bar plots represent the quantification of the technical replicates. (**b**) Left, representative Western blot showing protein levels of p70S6K1, p-p70S6K1 (Thr389) and FMRP. The molecular weight of each protein is indicated in kDa. Right, bar plots represent the quantification of the technical replicates. The quantification of FMRP results from the three detectable signals. (**c**) Left, representative Western blot showing protein levels of TSC1, TSC2 and RHEB. The molecular weight of each protein is indicated in kDa. Right, bar plots represent the quantification of the technical replicates. Error bars represent the standard error of the mean (n = 17-21 CTRL, 29-33 ASD). Each dot represents the average of at least two technical replicates per subject. Total proteins were normalised to the average of Coomassie staining and GAPDH. Phosphoproteins were normalised for respective total protein level.
